# Country-specific psychopharmacological risk of reporting suicidality comparing 38 antidepressants and lithium from the FDA Adverse Event Reporting System, 2017–2023

**DOI:** 10.3389/fpsyt.2024.1442490

**Published:** 2024-11-01

**Authors:** Andy Roger Eugene

**Affiliations:** Crisis Stabilization Unit, Larned State Hospital, Larned, KS, United States

**Keywords:** psychopharmacology, treatment-resistant depression, world health, suicide prevention, children, depression, drug selection, self-harm

## Abstract

**Background:**

The United States Food and Drug Administration (FDA) maintains a black-box warning for antidepressants warning of an increased risk of suicidality in children and young adults that is based on proprietary clinical trial data from study sponsors that were submitted for regulatory approval. This article aimed to assess whether the black-box warning for antidepressants is still valid today using recent drug safety data.

**Methods:**

Post-marketing adverse drug event data were obtained from the US FDA’s Adverse Event Reporting System (FAERS) for the years 2017 through 2023. Logistic regression analysis was conducted using the case versus non-case methodology and adjusted for gender, age group, drug role (primary drug, secondary drug, interacting drug, and concomitant drug), initial FDA reporting year, reporter country, and a drug*gene*age group interaction.

**Results:**

In the multivariate analysis, compared to fluoxetine and patients aged 25 to 64 years, children [adjusted reporting odds ratio (aROR) = 7.38, 95% CI, 6.02–9.05] and young adults (aROR = 3.49, 95% CI, 2.65–4.59) were associated with an increased risk of reporting suicidality, but not for the elderly (aROR = 0.76, 95% CI, 0.53–1.09). Relative to fluoxetine, esketamine was associated with the highest rate of reporting suicidality in children (aROR = 3.20, 95% CI, 2.25–4.54); however, esketamine was associated with a lower risk of reporting suicidality in young adults (aROR = 0.59, 95% CI, 0.41–0.84), but not significantly in the elderly (aROR = 0.77, 95% CI, 0.48–1.23). For country-specific findings, relative to the USA, the Slovak Republic, India, and Canada had the lowest risk of reporting suicidality. For the overall study population, desvenlafaxine (aROR = 0.61, 95% CI, 0.46–0.81) and vilazodone (aROR = 0.56, 95% CI, 0.32–0.99) were the only two antidepressants associated with a reduced risk of reporting suicidality.

**Conclusion:**

This study shows that with recent antidepressant drug safety data, the US FDA’s black-box warning for prescribing antidepressants to children and young adults is valid today in the USA. However, relative to the USA, 15 countries had a significantly lower risk of reporting suicidality, while 16 countries had a higher risk of reporting suicidality from 38 antidepressants and lithium.

## Introduction

Antidepressant drugs are among the most widely prescribed compounds in modern medicine (1). Antidepressants are prescribed by not only psychiatrists but also, in increasing numbers, general practitioners, family medicine specialists, obstetricians and gynecologists, internists, pediatricians, and other medical specialists due to screening campaigns recommended by the United States Preventive Services Task Force (2–5). Suicide rates have increased yearly since 1999, and recent data show an alarming rate of completed suicide in both the adolescent and adult populations in the USA (6). Among the adolescent population, the rates of American youth experiencing mental and behavioral health emergencies are so high that the American College of Emergency Physicians, the Emergency Nurses Association, and the American Academy of Pediatrics published a joint statement that provides guidance, resources, and strategies for emergency departments, healthcare systems, and communities who have youth experiencing mental and behavioral health emergencies (7). In their joint statement, the three clinical associations detail interventions using 1) school-based interventions, 2) community crisis response teams, 3) mobile response teams, and 4) diversion strategies for emergency medical services (7). Among the adult population, the US Centers for Disease Control and Prevention’s National Center for Injury Prevention and Control reports that from 2021 to 2022, the greatest increase in suicide deaths was found in persons ≥65 years of age at an 8.1% increase in suicide in the elderly population (8).

The US Food and Drug Administration (FDA) has maintained an antidepressant black-box warning since 2004 describing that antidepressants increase the risk of suicidality (ideation and behavior) in children and young adults up to 24 years old. Physicians from the FDA’s Division of Neuropharmacological Drug Products published a meta-analysis in 2006 analyzing randomized controlled placebo-controlled trials totaling 4,582 patients and concluded that antidepressant compounds in pediatric patients were associated with an increased risk of suicidality and self-harm (9). In young adults, another substantive analysis by the US FDA’s Center for Drug Evaluation and Research (CDER) reported that in a meta-analysis of 372 double-blind randomized placebo-controlled trials totaling 99,231 adult patients found an increased risk of suicidality in adults treated with antidepressants who were 24 years old and younger (10).

Further, Stone and colleagues reported that there was a reduced risk of suicidality in the elderly aged 65 years and greater (10). Of note, the analysis conducted by Hammad and colleagues, as well as Stone and colleagues, used a wide array of terms that encompass suicidality from clinical trial sponsors (9, 10). In the Hammad study, the included terms were as follows: suicide attempt, suicidal ideation, self-injury with intent unknown, injury events with not enough information to determine if represents self-injury or other type of injury, and lastly, preparatory actions toward imminent suicidal behavior (9). Similarly, the Stone study used the following terms from the clinical trial database submitted to the FDA to encompass suicidality: accident, asphyxiation, attempt, burn-, cut-, drown-, firearm, gas, gun, hang, hung, immolat-, injur-, jump, monoxide, mutilat-, overdos-, self damag-, self harm-, self inflict-, self injur-, shoot-, slash, suic-, poison-, and suffocation (10).

Considering the far-reaching public health impact of antidepressants and suicidality in the aforementioned age groups leading to a black-box warning since 2004, the natural question remains: is this black-box warning still valid today? Therefore, with this information as a background, the aim of this study was to 1) test the hypothesis that with recent pharmacovigilance data, children and young adults no longer have an increased risk of suicidality when treated with antidepressants; 2) specifically identify the antidepressants, if any, in the respective age groups that are protective and those associated with increased risk of suicidality; and 3) identify which countries had a higher risk as well as a reduced risk of reporting suicidality.

## Materials and methods

### Data source

Post-marketing adverse drug reaction (ADR) cases were obtained from the United States Food and Drug Administration’s Adverse Event Reporting System (FAERS) for each quarter from years 2017 through 2023, totaling 7 years (11). Patient names were already de-identified, and the unique case identifier (known as the “primaryid”) was used to link individual ASCII files delimited with the “$” sign that separates data fields within each of the seven available ASCII files, per quarter. Following the merging of the demographic data, patient cases were filtered to include only ages from 8 years to 120 years. Moreover, the following are the age-group categories used in this analysis as has been previously described: 8–17, 18–24, 25–65, and 65–120 (12).

### Clinical indications and antidepressant list

Patient cases were then only selected if clinical indications met a known psychiatric indication for prescribing antidepressants. The following is the list of clinical indications that met the study inclusion criteria: affective disorder, antidepressant therapy, anxiety, anxiety disorder, anxiolytic therapy, depressed mood, depression, depression suicidal, depressive symptom, generalized anxiety disorder, intentional overdose, intentional self-injury, major depression, obsessive-compulsive disorder, panic attack, panic disorder, perinatal depression, post-traumatic stress disorder, psychiatric symptom, self-destructive behavior, self-injurious ideation, social anxiety disorder, stress, suicidal ideation, and suicide attempt. A total of three antidepressant compounds, antidepressant combination products, and lithium were included in the antidepressant list. Lithium was chosen due to its well-known protective features in suicidality, which have been proven in multiple studies and various countries (13–16). The following is the list of antidepressants filtered from the general dataset and included in the study: fluoxetine (reference compound), agomelatine, amitriptyline, amoxapine, atomoxetine, bupropion, bupropion/dextromethorphan, bupropion/naltrexone (included despite having a primary indication for weight loss), citalopram, clomipramine, desipramine, desvenlafaxine, dothiepin, doxepin, duloxetine, escitalopram, esketamine, fluoxetine/olanzapine, fluvoxamine, imipramine, levomilnacipran, lithium, milnacipran, mirtazapine, nefazodone, nortriptyline, paroxetine, phenelzine, reboxetine, selegiline, sertraline, tianeptine, tranylcypromine, trazodone, trimipramine, venlafaxine, vilazodone, viloxazine, and vortioxetine.

### Data cleaning

Despite previous studies (17, 18) including missing age and gender data in the FAERS analysis, due to the widespread public health implications for this current study, any case missing the following information was removed from the final dataset: initial FDA reporting date, empty or unknown gender, missing drug role code, reported occupational code, and missing the preferred term outcome variables. Further, all duplicate cases were removed. To account for the prescribing of multiple antidepressants per unique case, the DRUGyyQq.TXT file’s drug role code was used to statistically adjust for whether the compound was a primary suspect drug, a secondary suspect drug, a concomitant drug, or an interacting drug. Further, to account for yearly trends in drug prescribing based on commercials, marketing of certain compounds, the COVID-19 pandemic, and other unforeseen influences on reporting of antidepressant ADRs, the initial FDA drug reporting year was also incorporated into the statistical model. Lastly, due to well-known country-specific differences in antidepressant prescribing and outcomes, the statistical model was adjusted to include a total of 108 countries and one country variable titled *country not specified* for a well-adjusted model.

### Statistics

The reporting odds ratio (ROR) with a corresponding 95% confidence interval showing the lower bound and upper bound was calculated using the case/non-case methodology via logistic regression analysis. The ROR signal is considered to be statistically significantly associated with the study outcome of risk of suicidality if the lower limit of the 95% confidence interval (CI) is greater than one, considered protective if the upper limit of the 95% CI is less than one, and not associated if the 95% CI crosses one. The final logistic regression model was adjusted for drug, age group, gender, drug role code, the initial FDA reporting year, country code (108 countries plus “country not specified”), and the drug*age group interaction for a robustly adjusted model. Fluoxetine and escitalopram are the only antidepressants approved by the US FDA for treating major depressive disorder in children with precedence of previous studies setting fluoxetine as the reference antidepressant; thus, fluoxetine was set as an active control for this current study (19, 20). Moreover, males were the reference gender, the age range 25–64 years was the reference age group, the primary suspect drug was the reference drug role (primary suspect drug, secondary suspect drug, concomitant drug, or an interacting drug), and the earliest year was established as the reference year. All statistical analyses and graphing were conducted using the R statistical programming language version 4.2.2 (The R Foundation for Statistical Computing, Vienna, Austria, https://www.R-project).

## Results

### Demographics and clinical indications

**Figure 1:**
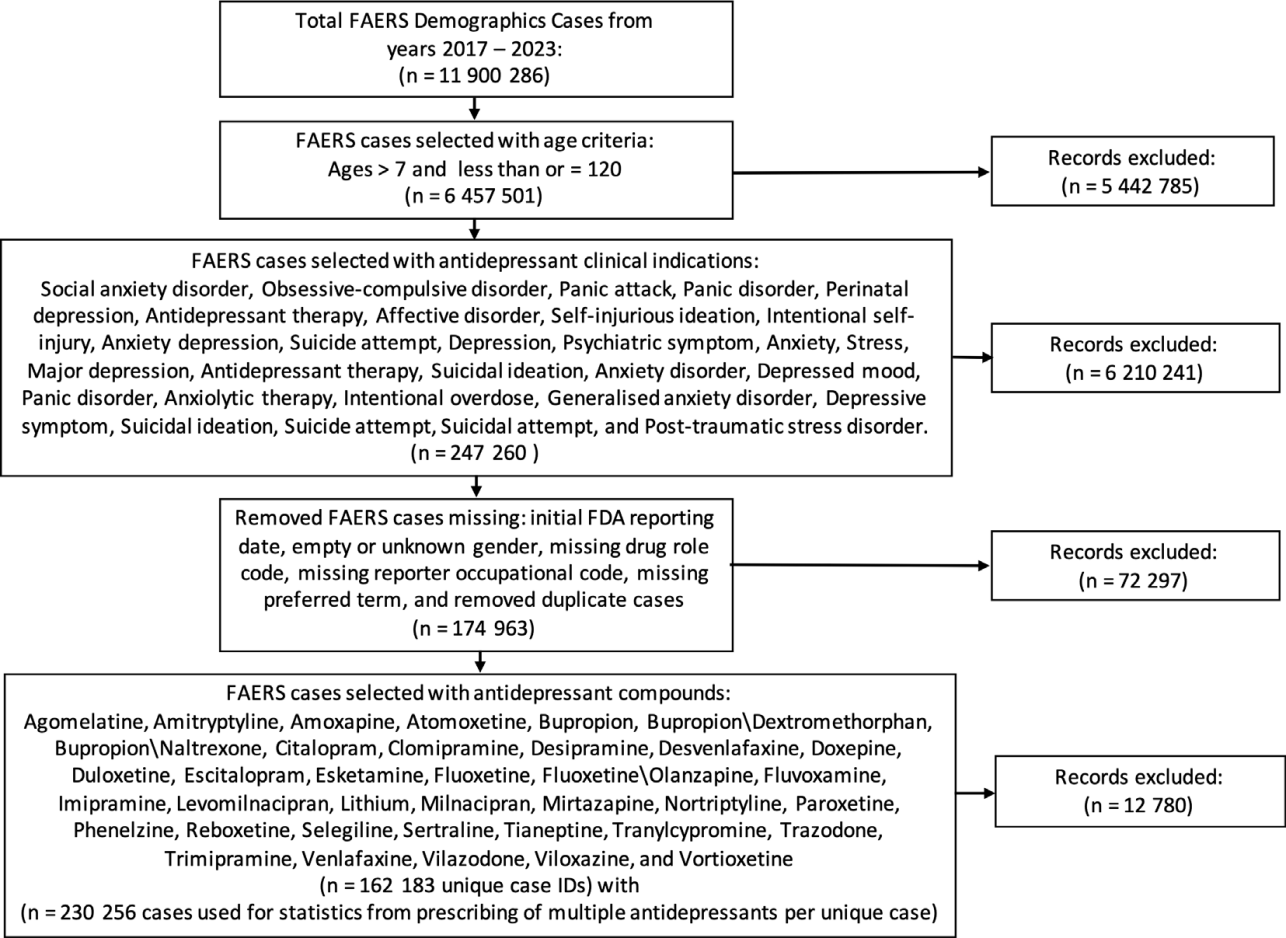
Study flow diagram of included cases from the FDA Adverse Event Reporting System from the years 2017 through 2023. These steps detail the inclusion criteria based on age range, clinical indication resulting in an antidepressant prescription, removal of incomplete data fields, and drugs of interest. FDA, Food and Drug Administration.

From the years 2017 through 2023, there were a total of 11,900,286 demographic cases that were later filtered and merged based on the study flow diagram inclusion process shown in **Figure 1**. After filtering the cases based on the study inclusion age range of 8–120 years, removing incomplete date fields, selecting FAERS cases with clinical indications using an antidepressant compound, and selecting cases with the pre-specified list of antidepressants, the final tally of unique patient case identifiers was 162,183 unique case, and a total of 230,256 cases were used in statistical calculations due to certain patients being prescribed more than one antidepressant. Female cases (n = 2,547) outnumbered male cases (n = 1,532), and the age group of 25–64 years had the most cases with 2,569, whereas the elderly population at the age range of 65–120 years (oldest identified age = 112) had the least number of cases of suicidality. The primary suspect drug role category had the greatest number of cases with 2,337 FAERS cases, and the drug–interaction drug role had the least number of FAERS cases with a total of 36 interactions. The final demographic information is shown in **Table 1**.

**Table 1:** Case versus non-case counts based on age groups, gender, and drug roles in the study.

	Cases (N = 4,079)	Non-cases (N = 158,104)	Total (N = 162,183)
Age, years, mean (SD, min–max)	40.3 (18.3, 8–95)	52.3 (18.8, 8–112)	
Age group			
(7–17)	470	5,712	6,182
(18–24)	599	9,046	9,645
(25–64)	2,569	98,325	100,894
(65–120)	441	45,021	45,462
Gender			
Male	1,532	49,363	50,895
Female	2,547	108,741	111,288
Drug role of concurrent antidepressants			
Primary suspect drug	2,337	58,252	60,589
Secondary suspect drug	2,203	60,220	62,423
Concomitant	1,254	99,491	100,745
Interacting	36	6,463	6,499
**Total drugs for statistical calculations**	5,830	224,426	230,256

There was a total of 25 clinical indications included in the study, and the top two clinical indications with the highest event rate for suicidality were suicide attempts (58.6% event rate) and suicidal ideations (29.5% event rate). In contrast, the two clinical indications with the least reported cases of suicidality and prescribed antidepressants were self-destructive behavior and self-injurious ideation, which had no reported cases of suicidality. The list of cases and non-cases based on clinical indication is shown in **Supplementary Table 1**.

### Overall population findings

From 2017 through 2023, the antidepressant with the highest number of reported cases of suicidality was esketamine (n = 846). There were several antidepressants with only one reported case of suicidality, identified as follows: bupropion/naltrexone (n = 1), desipramine (n = 1), nefazodone (n=1), and tianeptine (n = 1). In the overall study population, with male as the reference group for gender, female individuals were found to have a lower risk of reporting suicidality [adjusted reporting odds ratio (aROR) = 0.78, 95% CI, 0.74–0.83]. When adjusting the model for concurrently prescribed antidepressants, with reference being the primary suspect drug, antidepressants assigned the drug role of a secondary suspect compound (aROR = 1.00, 95% CI, 0.94–1.13) were not associated with an increased risk of reporting odds of suicidality. However, drug roles of concomitantly administered (aROR = 0.37, 95% CI, 0.34–0.40) and interacting (aROR = 0.18, 95% CI, 0.13–0.26) antidepressants had a lower risk of reporting suicidality. The initial FAERS reporting year (aROR = 1.11, 95% CI, 1.10–1.13) was associated with an 11% increased risk of reporting suicidality for 1 year in the final model.

In the multivariate analysis, with reference to fluoxetine, the only two compounds that were able to outperform fluoxetine with a reduced risk of reporting suicidality in the entire study population were found to be vilazodone (aROR = 0.56, 95% CI, 0.32–0.99) and desvenlafaxine (aROR = 0.61, 95% CI, 0.46–0.81). In contrast, in the adjusted model, when compared to fluoxetine, the following compounds were associated with an increased risk of reporting suicidality in the overall study population: venlafaxine (aROR = 1.33, 95% CI, 1.11–1.61), escitalopram (aROR = 1.48, 95% CI, 1.21–1.80), lithium (aROR = 1.54, 95% CI, 1.16–2.05), mirtazapine (aROR = 1.92, 95% CI, 1.57–2.35), vortioxetine (aROR = 1.98, 95% CI, 1.60–2.45), nortriptyline (aROR = 2.11, 95% CI, 1.41–3.17), trazodone (aROR = 2.45, 95% CI, 1.99–3.02), bupropion (aROR = 2.61, 95% CI, 2.17–3.15), esketamine (aROR = 2.75, 95% CI, 2.28–3.32), atomoxetine (aROR = 4.58, 95% CI, 2.92–7.19), and fluoxetine/olanzapine (aROR = 8.35, 95% CI, 1.94–35.90). When compared with fluoxetine, there was no difference in reporting risk of suicidality for the remaining antidepressants. **Supplementary Figure 1** shows the multivariate logistic regression results for this study, except for the interaction analysis component of the model evaluating the drug*age group interaction, which is provided in the results subsection below.

### Child and adolescent antidepressant outcomes

In the child and adolescent patients aged 8 to 17 years and treated with antidepressants, there was an increased risk of reporting suicidality (aROR = 7.38, 95% CI, 6.02–9.05), when compared to adults prescribed antidepressants between the ages of 25 years and 64 years. The antidepressant*age group interaction results showed that the only compound with an increased risk of reporting suicidality in children aged 8–17 years, relative to fluoxetine, was esketamine (aROR = 3.20, 95% CI, 2.25–4.54), as compared to treated adults aged 25–64 years. Relative to adults aged 25–64 years and treated with fluoxetine, the following antidepressants showed a reduced risk of reporting suicidality in children: amitriptyline (aROR = 0.38, 95% CI, 0.17–0.83), atomoxetine (aROR = 0.03, 95% CI, 0.66–0.13), bupropion (aROR = 0.27, 95% CI, 0.19–0.40), desvenlafaxine (aROR = 0.42, 95% CI, 0.20–0.89), duloxetine (aROR = 0.46, 95% CI, 0.25–0.85), escitalopram (aROR = 0.52, 95% CI, 0.37–0.73), lithium (aROR = 0.12, 95% CI, 0.55–0.28), mirtazapine (aROR = 0.43, 95% CI, 0.28–0.65), paroxetine (aROR = 0.58, 95% CI, 0.34–0.97), sertraline (aROR = 0.35, 95% CI, 0.26–0.46), trazodone (aROR = 0.49, 95% CI, 0.29–0.81), venlafaxine (aROR = 0.17, 95% CI, 0.10–0.30), vilazodone (aROR = 0.37, 95% CI, 0.005–0.29), and vortioxetine (aROR = 0.19, 95% CI, 0.11–0.34). The unmentioned interaction analysis results for the pediatric-aged patients did not have sufficient cases for a calculation or were not statistically different from the reference group.

### Young adult antidepressant outcomes

In comparison to the reference population of patients aged 25–years and treated with antidepressants, the young-adult patients aged 18–24 years had an increased risk of reporting suicidality when treated with antidepressants (aROR = 3.49, 95% CI, 2.65–4.59). When specifically investigating the details from the antidepressant*age group interaction, relative to being prescribed fluoxetine and aged 25–64 years, the following two antidepressants resulted in a higher risk of reporting suicidality in the 18- to 24-year-old patients: amitriptyline (aROR = 2.93, 95% CI, 1.79–4.80) and doxepin (aROR = 8.30, 95% CI, 2.90–2.38). The following antidepressants were found to have statistically significantly better performance for reducing suicidality relative to population aged 25–64 years and prescribed fluoxetine: atomoxetine (aROR = 0.91, 95% CI, 0.26–0.32), bupropion (aROR = 0.46, 95% CI, 0.32–0.68), duloxetine (aROR = 0.46, 95% CI, 0.23–0.92), esketamine (aROR = 0.59, 95% CI, 0.41–0.84), mirtazapine (aROR = 0.49, 95% CI, 0.32–0.76), paroxetine (aROR = 0.37, 95% CI, 0.21–0.66), sertraline (aROR = 0.53, 95% CI, 0.38–0.75), trazodone (aROR = 0.45, 95% CI, 0.28–0.73), venlafaxine (aROR = 0.56, 95% CI, 0.38–0.81), and vortioxetine (aROR = 0.66, 95% CI, 0.43–0.99). The unmentioned interactions for the young adult cases were not significant or did not have enough cases for statistical computations.

### Elderly antidepressant outcomes

Among the elderly patients aged 65–112 years, relative to patients aged 25–64 years and treated with antidepressants, there was no statistically significant difference in reporting suicidality (aROR = 0.76, 95% CI, 0.53–1.09). Further, relative to being prescribed fluoxetine, all of the following compounds showed a statistically significantly reduced risk of reporting suicidality in the elderly: bupropion (aROR = 0.46, 95% CI, 0.29–0.75), clomipramine (aROR = 0.98, 95% CI, 0.13–0.74), desvenlafaxine (aROR = 0.17, 95% CI, 0.59–0.50), escitalopram (aROR = 0.13, 95% CI, 0.69–0.25), mirtazapine (aROR = 0.59, 95% CI, 0.39–0.91), sertraline (aROR = 0.34, 95% CI, 0.21–0.54), trazodone (aROR = 0.49, 95% CI, 0.30–0.79), and venlafaxine (aROR = 0.36, 95% CI, 0.23–0.58). The remaining cases were not statistically significantly different from the reference groups or had an insufficient number of cases to compute the statistical results.

### Country-specific findings

**Figure 2:**
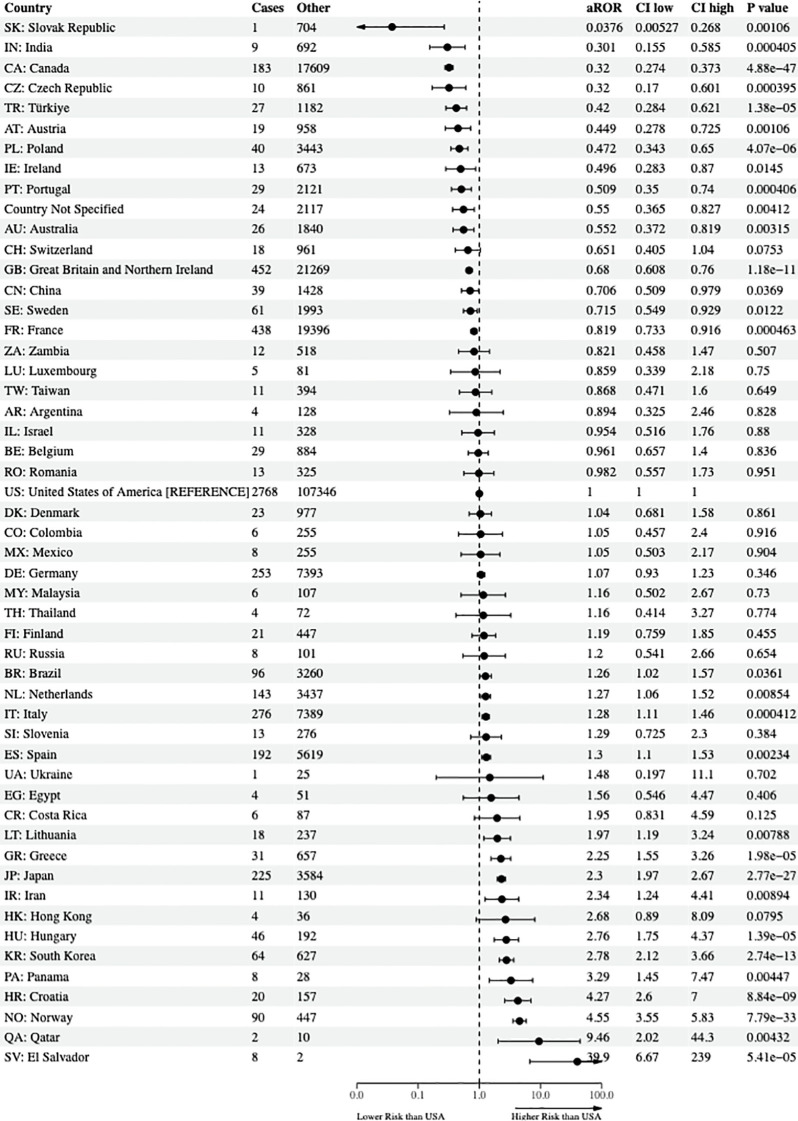
Risk of reporting suicidality among nations. The forest plots illustrate the adjusted reporting odds ratios (aROR) and 95% confidence intervals of nations reporting at least one reported case and non-case of suicidality to the US FDA Adverse Event Reporting System from the years 2017 through 2023 compared to the USA. FDA, Food and Drug Administration.

This study included a total of 108 countries worldwide, and one entry termed *country not specified* was included, per the FAERS documentation, resulting in 109 total entries for the reporter country in the final statistical model. The USA was set as the reference country and had the most reported cases of suicidality (cases = 2,768 and non-cases = 107,346). Relative to the USA, the following 15 countries had a significantly lower risk of reporting suicidality from the 38 antidepressants and lithium: the Slovak Republic (lowest), India, Canada, the Czech Republic, Turkey, Austria, Poland, Ireland, Portugal, *country not specified*, Australia, Great Britain and Northern Ireland, China, Sweden, and France. In contrast, the following 16 counties, in order of increasing aROR and relative to the USA, were found to have a statistically significantly higher risk of reporting suicidality: Brazil, Netherlands, Italy, Spain, Lithuania, Greece, Japan, Iran, Hungary, South Korea, Panama, Croatia, Norway, Qatar, and El Salvador (highest). The final results of the aROR for all nations—that is, even if the nation did not have a reported case of suicidality rendering the aROR incalculable—are shown in **Supplementary Table 2**. Due to the large number of countries in this study, **Figure 2** only illustrates the final country-specific psychopharmacological aROR for countries with at least one case and one non-case that were prescribed the 38 antidepressants and lithium.

## Discussion

The most important study findings are consistent with the US FDA’s results 20 years ago, leading to the black-box warning showing that there is an increased risk of suicidality in children and young adults treated with antidepressants (9, 10). Specifically, this study showed that relative to patients aged 25–64 years, children (aROR = 7.38, 95% CI, 6.02–9.05) and young adults (aROR = 3.49, 95% CI, 2.65–4.59) had an increased risk of reporting suicidality to the FDA Adverse Event Reporting System, as found from the CDER at the FDA using the meta-analysis procedure (9, 10). One inconsistent finding with previous studies is that in this analysis, the elderly age group was not associated with an increased risk of suicidality nor a decreased risk of suicidality (aROR = 0.82, 95% CI, 0.57–1.17). Further, there was a statistically significant increased risk of reporting suicidality by 253% in children treated with esketamine (aROR = 3.53, 95% CI, 2.51–4.98) in comparison to adults aged 25–64 years treated with fluoxetine. However, esketamine was associated with a lower risk of reporting suicidality in young adults (aROR = 0.69, 95% CI, 0.48–0.97), and there was no statistically significant difference in the elderly (aROR = 0.76, 95% CI, 0.53–1.09). McIntyre and colleagues recently conducted a pharmacovigilance study and also found that esketamine was associated with an increased reporting odds ratio of suicidal ideation (ROR = 7.58, 95% CI, 6.34–9.07) (21). This current study also found a 193% increased risk of reporting suicidality in young adults treated with amitriptyline (aROR = 2.93, 95% CI, 1.79–4.80) and a 730% increase in suicidality when prescribed doxepin (aROR = 8.30, 95% CI, 2.90–2.38), when compared to adults aged 25–64 years and treated with fluoxetine. This information helps with drug selection in high-risk suicidal patients but warrants further investigation as to the age-related pharmacodynamic interaction resulting in antidepressant-associated suicidality in pediatrics and the young-adult population relative to the general adult population.

Umetsu and colleagues published a pharmacovigilance analysis similar to this study by setting the age range of 25–64 years as a reference population but restricted the analysis to selective serotonin reuptake inhibitors (SSRIs), and they found by testing a similar interaction of antidepressant*age group that patients prescribed SSRIs and were less than 18 years old had the highest aROR of suicidality (aROR = 9.58, 95% CI, 8.97–10.23) (12). In another study, Li and colleagues conducted a meta-analysis of 17 observational studies in children and young adults between the ages of 5 years and 25 years, evaluating suicide or suicide attempt, and concluded that antidepressant use, especially SSRIs, increased the risk of suicidality in children and young adults, as with this current study (22). The FDA black-box warning for antidepressants in children and young adults does not preclude the prescribing of antidepressants but expresses caution that there should be a risk–benefit analysis in these patients.

Another critical finding is that, relative to fluoxetine, in the multivariate analysis, desvenlafaxine (aROR = 0.61, 95% CI, 0.46–0.81) and vilazodone (aROR = 0.56, 95% CI, 0.32–0.99) were comparably the most effective antidepressants overall in reducing the risk of suicidality in the entire study population. In pharmacogenomic testing of psychotropic drugs, desvenlafaxine is generally regarded as one of the go-to antidepressants in patients with two loss-of-function alleles comprising the phenotypic label of being a *CYP2D6* and/or *CYP2C19* poor metabolizer due to desvenlafaxine being metabolized mainly through conjugation and only sparsely through oxidative metabolism by cytochrome P450 enzymes (23). Similarly, according to the US FDA package insert, vilazodone is primarily metabolized by *CYP3A4* with only sparse contributions from the *CYP2D6* and *CYP2C19* genes (24). With this pharmacogenomic information, one may hypothesize that all *CYP3A4* antidepressant substrates are protective against suicidality. However, trazodone, which is metabolized by oxidative cleavage to the active metabolite via *CYP3A4*, was associated with an increased risk of suicidality, and levomilnacipran, which undergoes demethylation via *CYP3A4*, was not statistically significant from the active control antidepressant fluoxetine, a known strong *CYP2D6* and *CYP2C19* inhibitor (25–28).

Tiihonen and colleagues evaluated 10 antidepressants in a population of 15,390 patients, for an average follow-up period of 3.4 years, for the risks of completed suicide, suicide attempts, and mortality and reported that the highest risk of suicide was with venlafaxine, while the lowest risk of suicide was with fluoxetine (29). Continuing with venlafaxine, Barbui and colleagues conducted a systematic review and meta-analysis of the risk of suicide among SSRIs and found that venlafaxine and paroxetine resulted in an increased risk of suicide in adolescents, but there was no increased risk of suicide in the adult population, and they had a protective effect in the elderly population (30). In the current study, when compared to fluoxetine and the general adult population, venlafaxine was associated with a reduced risk of suicidality in children (aROR = 0.17, 95% CI, 0.10–0.30), young adults (aROR = 0.56, 95% CI, 0.38–0.81), and the elderly (aROR = 0.36, 95% CI, 0.23–0.58). For vilazodone, the current study results are consistent with the article of Thase and colleagues that assessed vilazodone-related treatment-emergent suicidal ideations using the Columbia Suicide Severity Rating Scale by category shift from no suicidal ideation and/or behavior to being present, and they found that less than 1% of patients treated with vilazodone experienced treatment-related adverse drug events (31).

In psychopharmacology, lithium is regarded as having anti-suicidal properties and has been well-reported in various regions throughout the world to be associated with a reduced risk of suicide (13–16). In this study, lithium (aROR = 1.54, 95% CI, 1.16–2.05) was associated with a higher risk of reporting suicidality, relative to the active control compound fluoxetine. This finding is similar to a study by Amsterdam and colleagues, who conducted a randomized double-blind study and found that fluoxetine outperformed lithium, and they calculated lithium as having a relapse rate that is 2.5 times higher than that of fluoxetine (32). In this current study, only the pediatric-age subgroup of cases of patients aged 8–17 years and treated with lithium (aROR = 0.12, 95% CI, 0.55–0.28) was protective with a lower risk of reporting suicidality; however, there was no statistical difference in young adults (aROR = 0.70, 95% CI, 0.38–1.28) or the elderly (aROR = 0.41, 95% CI, 0.16–1.02) prescribed lithium, relative to the general adult population and were prescribed the active control, fluoxetine. This is partially consistent with the study of Nabi and colleagues, who recently reported, in a meta-analysis of a total of 2,578 participants, that there was no statistically significant difference in the rates of suicidality when prescribed lithium (17). However, the meta-analysis comprised 113 million study participants in 2,678 regions throughout the world reported lithium in drinking water to be associated with reduced rates of suicidality (33). Taken together, to apply these findings to treatment-resistant depression, this current study’s results provide strong quantitative evidence that desvenlafaxine or vilazodone would be best recommended for all ages. Due to the increased risk of reporting suicidality in children and young adults when prescribed antidepressants, beyond psychopharmacological interventions in children and young adults experiencing depressive symptoms and/or anxiety, it is imperative to first resolve any psychosocial problems such as parental/guardian support, daily food security, affordable and safe housing, stable employment, drug and alcohol abstinence education and support groups, houses of worship, and loan repayment programs to resolve student-loan debt that may lead to thoughts of hopelessness and despair.

In terms of country-specific psychopharmacological suicidality findings, as shown in **Figure 2**, the Slovak Republic (aROR = 0.04, 95% CI, 0.005–0.27) and India (aROR = 0.301, 95% CI, 0.155–0.585) had the lowest risk of reporting suicidality when prescribed the 38 antidepressants and lithium in their population when referenced to the USA. With respect to study outcomes relative to geographic proximity to the USA, this study showed that Canada (aROR = 0.32, 95% CI, 0.274–0.373), ranked third in countries with the lowest risk of reporting suicidality and stationed north of mainland USA, had a protective effect of the antidepressants. Mexico (aROR = 1.05, 95% CI, 0.50–2.17), which is located geographically south of the mainland USA, did not have a statistically significant difference in risk of reporting suicidality from the 38 antidepressants and lithium. To over-simplify, the complex matters leading to the varied differences in suicidality outcomes between nations may range from cultural differences in parental support, guardian support, family cohesion, and pharmacogenomic influences, as well as remaining single versus the well-documented protective effect of being married; however, from a healthcare perspective, many nations have universal healthcare for its citizens, whereas others do not (34–38). This overly simplistic factor alone may result in improved mental health outcomes so that patients would not have to fear financial bankruptcy from seeking initial healthcare appointments, follow-up appointments, and being able to afford high-cost medications (39, 40). Lastly, the results of this study are consistent with the findings of Whitely and colleagues in Australia, who found that antidepressants increased the risk of suicide and self-harm in children, adolescents, and young adults (41). However, from a country outcomes perspective, Australia (aROR = 0.55, 95% CI, 0.37–0.82) was associated with a reduced risk of reporting suicidality, relative to the USA.

### Limitations

A fundamental limitation with disproportionality analyses, case versus non-case studies, as with case–control studies is that the association of a drug with the outcome variable of suicidality does not imply that the drug actually caused the outcome. Further, as with all databases, many cases may be incomplete as well as duplicated in the reporting of the medical personnel, pharmacies, and caregivers. Moreover, the occurrence rate of the drug of interest with the FAERS preferred terms adverse events does not estimate the incidence of the adverse drug event. Despite these limitations, the FAERS database provides access to recent as well as historic drug data that are invaluable in psychopharmacology, and the results of this study set the stage for informing psychopharmacology practice guidelines, especially in treatment-resistant depression as well as in treating high-risk suicidal patients.

## Conclusions

This population-based post-marketing surveillance study affirms the original findings of the US FDA leading to the black-box warning for prescribing antidepressants in children and young adults. This study also found that out of a total of 38 antidepressants and lithium, only desvenlafaxine and vilazodone—both *CYP3A4* substrates—were associated with a reduced risk of reporting suicidality in the entire study population. The two clinical indications with patients who were prescribed antidepressants, self-destructive behavior and self-injurious ideation, did not have any reported cases of suicidality. Lastly, this study also showed significant differences in country-specific outcomes of suicidality from the 38 antidepressants and lithium, even with geographically neighboring countries. Overall, this study provides population-based evidence to inform clinical practice guidelines and decision-making in treatment-resistant depression, post-traumatic stress disorder, anxiety, and various clinical conditions resulting in the prescribing of antidepressant compounds.

## Data Availability

Publicly available datasets were analyzed in this study. This data can be found here: https://fis.fda.gov/extensions/FPD-QDE-FAERS/FPD-QDE-FAERS.html.

## Supplementary material

Supplementary Figure 1 Risk of reporting suicidality among 38 antidepressants and lithium. The forest plots illustrate the adjusted reporting odds-ratios (aROR) and 95% confidence intervals of the 38 antidepressants and lithium, gender, and drug role relative to the active control fluoxetine. The adjustments made for the age-group*drug interaction and reporter country are not shown and provided in the text and supplementary tables, respectively. The results are based on data from U.S. FDA Adverse Event Reporting System from the years 2017 through 2023.

## References

[1] Brody DJ. Antidepressant Use Among Adults: United States, 2015-2018 (2022). NCHS Data Brief No 377. Available online at: https://www.cdc.gov/nchs/products/databriefs/db377.htm?ref=assuma-o-controle-de-sua-saude.com (Accessed May 5, 2024). 33054926

[2] US Preventive Services Task Force Barry MJ Nicholson WK Silverstein M Chelmow D Coker TR. Screening for depression and suicide risk in adults: US preventive services task force recommendation statement. JAMA. (2023) 329:2057–67. doi: 10.1001/jama.2023.9297 37338872

[3] US Preventive Services Task Force Mangione CM Barry MJ Nicholson WK Cabana M Chelmow D. Screening for depression and suicide risk in children and adolescents: US preventive services task force recommendation statement. JAMA. (2022) 328:1534–42. doi: 10.1001/jama.2022.16946 36219440

[4] Curry SJ Krist AH Owens DK Barry MJ Caughey AB Davidson KW. Screening for intimate partner violence, elder abuse, and abuse of vulnerable adults: US preventive services task force final recommendation statement. JAMA. (2018) 320:1678–87. doi: 10.1001/jama.2018.14741 30357305

[5] US Preventive Services Task Force Barry MJ Nicholson WK Silverstein M Coker TR Davidson KW. Screening for anxiety disorders in adults: US preventive services task force recommendation statement. JAMA. (2023) 329:2163–70. doi: 10.1001/jama.2023.9301 37338866

[6] Stone DM Mack KA Qualters J. Notes from the field: recent changes in suicide rates, by race and ethnicity and age group - United States, 2021. MMWR Morb Mortal Wkly Rep. (2023) 72:160–2. doi: 10.15585/mmwr.mm7206a4 PMC9925140 36757870

[7] Saidinejad M Duffy S Wallin D Hoffmann JA Joseph MM Uhlenbrock JS. The management of children and youth with pediatric mental and behavioral health emergencies. Pediatrics. (2023). doi: 10.1542/peds.2023-063255 37581617

[8] Centers for Disease Control and Prevention, NC for IP and C. Suicide Data and Statistics | Suicide | CDC . Available online at: https://www.cdc.gov/suicide/suicide-data-statistics.html (Accessed September 10, 2023).

[9] Hammad TA Laughren T Racoosin J. Suicidality in pediatric patients treated with antidepressant drugs. Arch Gen Psychiatry. (2006) 63:332–9. doi: 10.1001/archpsyc.63.3.332 16520440

[10] Stone M Laughren T Jones ML Levenson M Holland PC Hughes A. Risk of suicidality in clinical trials of antidepressants in adults: analysis of proprietary data submitted to US Food and Drug Administration. BMJ. (2009) 339:b2880. doi: 10.1136/bmj.b2880 19671933 PMC2725270

[11] US Food and Drug Administration. FAERS Quarterly Data Extract Files . Available online at: https://fis.fda.gov/extensions/FPD-QDE-FAERS/FPD-QDE-FAERS.html (Accessed May 1, 2024).

[12] Umetsu R Abe J Ueda N Kato Y Matsui T Nakayama Y. Association between selective serotonin reuptake inhibitor therapy and suicidality: analysis of U.S. Food and drug administration adverse event reporting system data. Biol Pharm Bull. (2015) 38:1689–99. doi: 10.1248/bpb.b15-00243 26521821

[13] Dawson EB Moore TD McGanity WJ. The mathematical relationship of drinking water lithium and rainfall to mental hospital admission. Dis Nerv Syst. (1970) 31:811–20. 5497853

[14] Giotakos O Nisianakis P Tsouvelas G Giakalou V-V. Lithium in the public water supply and suicide mortality in Greece. Biol Trace Elem Res. (2013) 156:376–9. doi: 10.1007/s12011-013-9815-4 24072668

[15] Kapusta ND Mossaheb N Etzersdorfer E Hlavin G Thau K Willeit M. Lithium in drinking water and suicide mortality. Br J Psychiatry. (2011) 198:346–50. doi: 10.1192/bjp.bp.110.091041 21525518

[16] Schrauzer GN Shrestha KP. Lithium in drinking water and the incidences of crimes, suicides, and arrests related to drug addictions. Biol Trace Elem Res. (1990) 25:105–13. doi: 10.1007/BF02990271 1699579

[17] Nabi Z Stansfeld J Plöderl M Wood L Moncrieff J. Effects of lithium on suicide and suicidal behaviour: a systematic review and meta-analysis of randomised trials. Epidemiol Psychiatr Sci. (2022) 31:e65. doi: 10.1017/S204579602200049X 36111461 PMC9533115

[18] Zhang L Mao W Liu D Hu B Lin X Ran J. Risk factors for drug-related acute pancreatitis: an analysis of the FDA adverse event reporting system (FAERS). Front Pharmacol. (2023) 14:1231320. doi: 10.3389/fphar.2023.1231320 38044938 PMC10690789

[19] Jansen JP Fleurence R Devine B Itzler R Barrett A Hawkins N. Interpreting indirect treatment comparisons and network meta-analysis for health-care decision making: report of the ISPOR Task Force on Indirect Treatment Comparisons Good Research Practices: part 1. Value Health. (2011) 14:417–28. doi: 10.1016/j.jval.2011.04.002 21669366

[20] Cooper WO Callahan ST Shintani A Fuchs DC Shelton RC Dudley JA. Antidepressants and suicide attempts in children. Pediatrics. (2014) 133:204–10. doi: 10.1542/peds.2013-0923 PMC3904271 24394688

[21] McIntyre RS Mansur RB Rosenblat JD Teopiz KM Kwan ATH. The association between ketamine and esketamine and suicidality: reports to the Food And Drug Administration Adverse Event Reporting System (FAERS). Expert Opin Drug Saf. (2024), 1–6. doi: 10.1080/14740338.2024.2368827 38884147

[22] Li K Zhou G Xiao Y Gu J Chen Q Xie S. Risk of suicidal behaviors and antidepressant exposure among children and adolescents: A meta-analysis of observational studies. Front Psychiatry. (2022) 13:880496. doi: 10.3389/fpsyt.2022.880496 35693956 PMC9178080

[23] Calleja S Zubiaur P Ochoa D Villapalos-García G Mejia-Abril G Soria-Chacartegui P. Impact of polymorphisms in CYP and UGT enzymes and ABC and SLCO1B1 transporters on the pharmacokinetics and safety of desvenlafaxine. Front Pharmacol. (2023) 14:1110460. doi: 10.3389/fphar.2023.1110460 36817149 PMC9934922

[24] Allergan Inc. DailyMed - VIIBRYD- vilazodone hydrochloride tablet VIIBRYD- vilazodone hydrochloride kit(2023). Available online at: https://www.dailymed.nlm.nih.gov/dailymed/drugInfo.cfm?setid=4c55ccfb-c4cf-11df-851a-0800200c9a66 (Accessed October 5, 2024).

[25] Teva Pharmaceuticals USA Inc. DailyMed - TRAZODONE HYDROCHLORIDE tablet(2021). Available online at: https://www.dailymed.nlm.nih.gov/dailymed/drugInfo.cfm?setid=9c4da6ad-d4d1-4e8c-918d-8ac48512a93f (Accessed October 5, 2024).

[26] Allergan Inc. DailyMed - FETZIMA- levomilnacipran hydrochloride capsule, extended release FETZIMA- levomilnacipran hydrochloride kit(2024). Available online at: https://www.dailymed.nlm.nih.gov/dailymed/drugInfo.cfm?setid=f371258d-91b3-4b6a-ac99-434a1964c3af (Accessed October 5, 2024).

[27] Dista Products Company. DailyMed - PROZAC- fluoxetine hydrochloride capsule(2023). Available online at: https://www.dailymed.nlm.nih.gov/dailymed/drugInfo.cfm?setid=c88f33ed-6dfb-4c5e-bc01-d8e36dd97299 (Accessed October 5, 2024).

[28] US Food and Drug Administration. Drug development and drug interactions | Table of substrates, inhibitors and inducers | FDA(2023). Available online at: https://www.fda.gov/drugs/drug-interactions-labeling/drug-development-and-drug-interactions-table-substrates-inhibitors-and-inducers (Accessed October 4, 2024).

[29] Tiihonen J Lönnqvist J Wahlbeck K Klaukka T Tanskanen A Haukka J. Antidepressants and the risk of suicide, attempted suicide, and overall mortality in a nationwide cohort. Arch Gen Psychiatry. (2006) 63:1358–67. doi: 10.1001/archpsyc.63.12.1358 17146010

[30] Barbui C Esposito E. Cipriani A. Selective serotonin reuptake inhibitors and risk of suicide: a systematic review of observational studies. CMAJ. (2009) 180:291–7. doi: 10.1503/cmaj.081514 PMC2630355 19188627

[31] Thase ME Edwards J Durgam S Chen C Chang C-T Mathews M. Effects of vilazodone on suicidal ideation and behavior in adults with major depressive disorder or generalized anxiety disorder: *post-hoc* analysis of randomized, double-blind, placebo-controlled trials. Int Clin Psychopharmacol. (2017) 32:281–8. doi: 10.1097/YIC.0000000000000180 PMC5540350 28538024

[32] Amsterdam JD Shults J. Efficacy and safety of long-term fluoxetine versus lithium monotherapy of bipolar II disorder: a randomized, double-blind, placebo-substitution study. Am J Psychiatry. (2010) 167:792–800. doi: 10.1176/appi.ajp.2009.09020284 20360317 PMC2896440

[33] Eyre-Watt B Mahendran E Suetani S Firth J Kisely S Siskind D. The association between lithium in drinking water and neuropsychiatric outcomes: A systematic review and meta-analysis from across 2678 regions containing 113 million people. Aust N Z J Psychiatry. (2021) 55:139–52. doi: 10.1177/0004867420963740 33045847

[34] Harris TL Molock SD. Cultural orientation, family cohesion, and family support in suicide ideation and depression among African American college students. Suicide Life-Threatening Behav. (2000) 30:341–53. doi: 10.1111/j.1943-278X.2000.tb01100.x 11210059

[35] Macalli M Tournier M Galéra C Montagni I Soumare A Côté SM. Perceived parental support in childhood and adolescence and suicidal ideation in young adults: a cross-sectional analysis of the i-Share study. BMC Psychiatry. (2018) 18:373. doi: 10.1186/s12888-018-1957-7 30482174 PMC6260717

[36] LeCloux M Maramaldi P Thomas K Wharff E. Family support and mental health service use among suicidal adolescents. J Child Fam Stud. (2016) 25:2597–606. doi: 10.1007/s10826-016-0417-6

[37] Campisi SC Carducci B Akseer N Zasowski C Szatmari P Bhutta ZA. Suicidal behaviours among adolescents from 90 countries: a pooled analysis of the global school-based student health survey. BMC Public Health. (2020) 20:1102. doi: 10.1186/s12889-020-09209-z 32772922 PMC7416394

[38] Abio A Owusu PN Posti JP Bärnighausen T Shaikh MA Shankar V. Cross-national examination of adolescent suicidal behavior: a pooled and multi-level analysis of 193,484 students from 53 LMIC countries. Soc Psychiatry Psychiatr Epidemiol. (2022) 57:1603–13. doi: 10.1007/s00127-022-02287-x PMC9288956 35445842

[39] Kalousova L Burgard SA. Debt and foregone medical care. J Health Soc Behav. (2013) 54:204–20. doi: 10.1177/0022146513483772 23620501

[40] Naranjo DE Glass JE Williams EC. Persons with debt burden are more likely to report suicide attempt than those without: A national study of US adults. J Clin Psychiatry. (2021) 82:31989. doi: 10.4088/JCP.19m13184 33989465

[41] Whitely M Raven M Jureidini J. Antidepressant prescribing and suicide/self-harm by young Australians: regulatory warnings, contradictory advice, and long-term trends. Front Psychiatry. (2020) 11:478. doi: 10.3389/fpsyt.2020.00478 32587531 PMC7299202

