# Supplementary material for: Country-specific psychopharmacological risk of reporting suicidality comparing 38 antidepressants and lithium from the FDA Adverse Event Reporting System, 2017–2023
Source: Front Psychiatry. 2024 Nov 1;15:1442490. doi: 10.3389/fpsyt.2024.1442490 (PMC11580034; doi:10.3389/fpsyt.2024.1442490)
Supplement: Supplementary Figure 1 — Risk of reporting suicidality among 38 antidepressants and lithium. The forest plots illustrate the adjusted reporting odds-ratios (aROR) and 95% confidence intervals of the 38 antidepressants and lithium, gender, and drug role relative to the active control fluoxetine. The adjustments made for the age-group*drug interaction and reporter country are not shown and provided in the text and supplementary tables, respectively. The results are based on data from U.S. FDA Adverse Event Reporting System from the years 2017 through 2023. [file Image1.pdf]

| Compound | Cases | Other | aROR | CI low | CI high | P value |
|---|---|---|---|---|---|---|
| Viloxazine | 2 | 30 | 1.13e–06 | 0 | | 0.99 |
| Reboxetine | 0 | 131 | 1.27e–06 | 0 | | 0.975 |
| Levomilnacipran | 4 | 398 | 1.39e–06 | 5.13e–198 | 3.76e+185 | 0.952 |
| Nefazodone | 1 | 156 | 1.4e–06 | 0 | | 0.976 |
| Milnacipran | 7 | 419 | 1.85e–06 | 2.26e–194 | 1.52e+182 | 0.952 |
| Tianeptine | 1 | 268 | 0.29 | 0.0401 | 2.1 | 0.22 |
| Bupropion\Naltrexone | 1 | 268 | 0.297 | 0.041 | 2.15 | 0.229 |
| Bupropion\Dextromethorphan | 2 | 167 | 0.48 | 0.117 | 1.96 | 0.307 |
| Vilazodone | 16 | 2019 | 0.557 | 0.315 | 0.985 | 0.0442 |
| Desvenlafaxine | 92 | 8239 | 0.611 | 0.459 | 0.813 | 0.00071 |
| Phenelzine | 9 | 657 | 0.644 | 0.3 | 1.38 | 0.26 |
| Trimipramine | 6 | 268 | 0.907 | 0.284 | 2.89 | 0.868 |
| Duloxetine | 224 | 17768 | 0.93 | 0.744 | 1.16 | 0.529 |
| Fluvoxamine | 34 | 1625 | 0.958 | 0.56 | 1.64 | 0.875 |
| Fluoxetine [REFERENCE] | 562 | 17647 | 1 | 1 | 1 | |
| Paroxetine | 202 | 12790 | 1 | 0.793 | 1.27 | 0.977 |
| Sertraline | 682 | 35497 | 1.09 | 0.901 | 1.32 | 0.374 |
| Amitryptyline | 163 | 8402 | 1.19 | 0.913 | 1.55 | 0.196 |
| Clomipramine | 39 | 2205 | 1.2 | 0.814 | 1.75 | 0.363 |
| Imipramine | 5 | 440 | 1.27 | 0.464 | 3.49 | 0.639 |
| Venlafaxine | 559 | 26517 | 1.33 | 1.11 | 1.61 | 0.00238 |
| Agomelatine | 9 | 758 | 1.35 | 0.678 | 2.69 | 0.393 |
| Desipramine | 1 | 97 | 1.35 | 0.184 | 9.88 | 0.768 |
| Tranylcypromine | 7 | 281 | 1.35 | 0.587 | 3.11 | 0.48 |
| Doxepine | 34 | 1555 | 1.4 | 0.864 | 2.27 | 0.171 |
| Escitalopram | 451 | 20240 | 1.48 | 1.21 | 1.8 | 0.000137 |
| Lithium | 102 | 4646 | 1.54 | 1.16 | 2.05 | 0.00303 |
| Dothiepin | 2 | 177 | 1.77 | 0.429 | 7.31 | 0.429 |
| Mirtazapine | 453 | 15689 | 1.92 | 1.57 | 2.35 | 1.48e–10 |
| Amoxapine | 3 | 54 | 1.94 | 0.452 | 8.3 | 0.374 |
| Citalopram | 2 | 104 | 1.98 | 0.266 | 14.7 | 0.505 |
| Vortioxetine | 324 | 6992 | 1.98 | 1.6 | 2.45 | 3.36e–10 |
| Selegiline | 7 | 285 | 2.1 | 0.967 | 4.58 | 0.0608 |
| Nortriptyline | 34 | 1690 | 2.11 | 1.41 | 3.17 | 0.000283 |
| Trazodone | 318 | 11845 | 2.45 | 1.99 | 3.02 | 4.62e–17 |
| Bupropion | 594 | 16521 | 2.61 | 2.17 | 3.15 | 7.78e–24 |
| Esketamine | 846 | 6987 | 2.75 | 2.28 | 3.32 | 8.3e–26 |
| Atomoxetine | 30 | 565 | 4.58 | 2.92 | 7.19 | 3.63e–11 |
| Fluoxetine\Olanzapine | 2 | 29 | 8.35 | 1.94 | 35.9 | 0.00431 |
| GENDER | | | | | | |
| Male [REFERENCE] | 2180 | 68860 | 1 | 1 | 1 | |
| Female | 3650 | 155566 | 0.78 | 0.737 | 0.825 | 5.18e–18 |
| DRUG ROLE | | | | | | |
| Primary Suspect Drug [REFERENCE] | 2337 | 58252 | 1 | 1 | 1 | |
| Secondary Suspect Drug | 2203 | 60220 | 1 | 0.942 | 1.07 | 0.915 |
| Concomitant | 1254 | 99491 | 0.372 | 0.344 | 0.402 | 7.59e–136 |
| Interacting | 36 | 6463 | 0.184 | 0.132 | 0.258 | 3.73e–23 |
| Initial FDA Reporting Year (for 1 year) | | | 1.11 | 1.1 | 1.13 | 4.79e–51 |
| AGE GROUP | | | | | | |
| Age Group: 25–64 [REFERENCE] | 3543 | 144058 | 1 | 1 | 1 | |
| Age Group: 8–17 | 935 | 8609 | 7.38 | 6.02 | 9.05 | 3.84e–82 |
| Age Group: 18–24 | 811 | 12841 | 3.49 | 2.65 | 4.59 | 4.06e–19 |
| Age Group: 65–120 | 541 | 58918 | 0.762 | 0.533 | 1.09 | 0.136 |

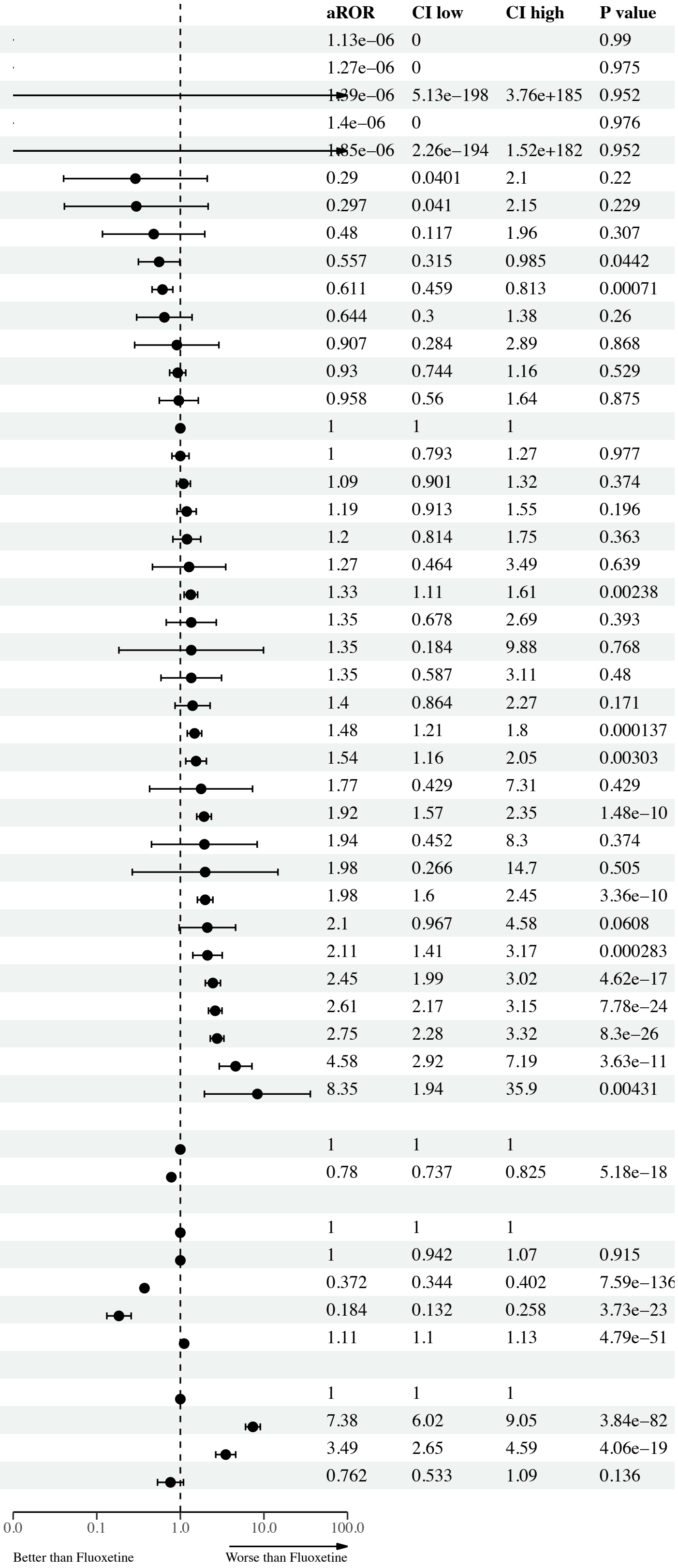


**Supplementary Figure 1: Risk of reporting suicidality among 38 antidepressants and lithium**.
The forest plots illustrate the adjusted reporting odds-ratios (aROR) and 95% confidence intervals of the 38 antidepressants and lithium, gender, and drug role relative to the active control fluoxetine. The adjustments made for the age-group*drug interaction and reporter country are not shown and provided in the text and supplementary tables, respectively. The results are based on data from U.S. FDA Adverse Event Reporting System from the years 2017 through 2023.
