# Supplementary material for: Country-specific psychopharmacological risk of reporting suicidality comparing 38 antidepressants and lithium from the FDA Adverse Event Reporting System, 2017–2023
Source: Front Psychiatry. 2024 Nov 1;15:1442490. doi: 10.3389/fpsyt.2024.1442490 (PMC11580034; doi:10.3389/fpsyt.2024.1442490)
Supplement: Supplementary file 2 [file Table1.docx]

| Supplementary Table 1: Case count and event rates based on clinical indications of patients prescribed antidepressants and not prescribed antidepressants. | | | |
| --- | --- | --- | --- |
|  | **Cases**  **(N = 5830)** | **Non-Cases**  **(N = 224 426)** | **Event-Rate (%)** |
| Affective disorder | 42 | 5653 | 0.7 |
| Antidepressant therapy | 68 | 6293 | 1.1 |
| Anxiety | 623 | 51940 | 1.2 |
| Anxiety disorder | 16 | 2578 | 0.6 |
| Anxiolytic therapy | 6 | 940 | 0.6 |
| Depressed mood | 34 | 2516 | 1.4 |
| Depression | 2239 | 115406 | 1.9 |
| Depression suicidal | 18 | 668 | 2.7 |
| Depressive symptom | 39 | 1403 | 2.8 |
| Generalised anxiety disorder | 23 | 2725 | 0.8 |
| Intentional overdose | 139 | 1091 | 12.7 |
| Intentional self-injury | 65 | 605 | 10.7 |
| Major depression | 649 | 15183 | 4.3 |
| Obsessive-compulsive disorder | 60 | 3770 | 1.6 |
| Panic attack | 27 | 2509 | 1.1 |
| Panic disorder | 12 | 1813 | 0.7 |
| Perinatal depression | 2 | 440 | 0.5 |
| Post-traumatic stress disorder | 31 | 3525 | 0.9 |
| Psychiatric symptom | 35 | 358 | 9.8 |
| Self-destructive behaviour | 0 | 14 | 0.0 |
| Self-injurious ideation | 0 | 41 | 0.0 |
| Social anxiety disorder | 9 | 537 | 1.7 |
| Stress | 50 | 1098 | 4.6 |
| Suicidal ideation | 307 | 1041 | 29.5 |
| Suicide attempt | 1336 | 2279 | 58.6 |
