# Supplementary material for: Country-specific psychopharmacological risk of reporting suicidality comparing 38 antidepressants and lithium from the FDA Adverse Event Reporting System, 2017–2023
Source: Front Psychiatry. 2024 Nov 1;15:1442490. doi: 10.3389/fpsyt.2024.1442490 (PMC11580034; doi:10.3389/fpsyt.2024.1442490)
Supplement: Supplementary file 3 [file Table2.docx]

| Supplementary Table 2: Adjusted Reporting Odds Ratios for 108 countries and on entry for *Country Not Specified* based on the International Organization for Standardization (ISO) 3166 standard for FAERS data from 2017 to 2023. | | | | | | |
| --- | --- | --- | --- | --- | --- | --- |
| Country | **Cases** | **Non-Cases** | **aROR** | **95% CI low** | **95% CI high** | **P value** |
| US: United States of America [REFERENCE] | 2768 | 107346 | 1 | 1 | 1 |  |
| AE: United Arab Emirates | 0 | 31 | 0.000000741 | 0 | Infinity | 0.983 |
| AF: Afghanistan | 0 | 7 | 0.00000232 | 0 | Infinity | 0.993 |
| AL: Albania | 0 | 9 | 0.000000293 | 0 | Infinity | 0.991 |
| AR: Argentina | 4 | 128 | 0.894 | 0.325 | 2.46 | 0.828 |
| AT: Austria | 19 | 958 | 0.449 | 0.278 | 0.725 | 0.00106 |
| AU: Australia | 26 | 1840 | 0.552 | 0.372 | 0.819 | 0.00315 |
| AW: Aruba | 0 | 4 | 0.00000124 | 0 | Infinity | 0.995 |
| BA: Bosnia and Herzegovina | 1 | 0 | 896000000 | 0 | Infinity | 0.996 |
| BB: Barbados | 0 | 1 | 0.000000517 | 0 | Infinity | 0.997 |
| BE: Belgium | 29 | 884 | 0.961 | 0.657 | 1.4 | 0.836 |
| BG: Bulgaria | 0 | 53 | 0.0000007 | 0 | Infinity | 0.979 |
| BH: Bahrain | 0 | 9 | 0.000000281 | 0 | Infinity | 0.991 |
| BR: Brazil | 96 | 3260 | 1.26 | 1.02 | 1.57 | 0.0361 |
| CA: Canada | 183 | 17609 | 0.32 | 0.274 | 0.373 | 4.88E-47 |
| CH: Switzerland | 18 | 961 | 0.651 | 0.405 | 1.04 | 0.0753 |
| CL: Chile | 0 | 93 | 0.00000127 | 0 | Infinity | 0.972 |
| CN: China | 39 | 1428 | 0.706 | 0.509 | 0.979 | 0.0369 |
| CO: Colombia | 6 | 255 | 1.05 | 0.457 | 2.4 | 0.916 |
| Country Not Specified | 24 | 2.12E+03 | 0.55 | 0.365 | 0.827 | 0.00412 |
| CR: Costa Rica | 6 | 87 | 1.95 | 0.831 | 4.59 | 0.125 |
| CU: Cuba | 0 | 4 | 0.0000011 | 0 | Infinity | 0.994 |
| CY: Cyprus | 0 | 42 | 0.000000685 | 0 | Infinity | 0.981 |
| CZ: Czech Republic | 10 | 861 | 0.32 | 0.17 | 0.601 | 0.000395 |
| DE: Germany | 253 | 7393 | 1.07 | 0.93 | 1.23E+00 | 0.346 |
| DK: Denmark | 23 | 977 | 1.04 | 0.681 | 1.58 | 0.861 |
| DO: Dominican Republic | 0 | 4 | 0.00000103 | 0 | Infinity | 0.994 |
| DZ: Algeria | 0 | 1 | 0.000000861 | 0 | Infinity | 0.997 |
| EC: Ecuador | 0 | 22 | 0.000000286 | 0 | Infinity | 0.985 |
| EE: Estonia | 0 | 35 | 0.00000108 | 0 | Infinity | 0.983 |
| EG: Egypt | 4 | 51 | 1.56 | 0.546 | 4.47 | 0.406 |
| ES: Spain | 192 | 5619 | 1.3 | 1.1 | 1.53 | 0.00234 |
| ET: Ethiopia | 0 | 1 | 0.000000772 | 0 | Infinity | 0.997 |
| FI: Finland | 21 | 447 | 1.19 | 0.759 | 1.85 | 0.455 |
| FR: France | 438 | 19396 | 0.819 | 0.733 | 0.916 | 0.000463 |
| GB: Great Britain and Northern Ireland | 452 | 21269 | 0.68 | 0.608 | 0.76 | 1.18E-11 |
| GE: Georgia | 0 | 2 | 0.000000126 | 0 | Infinity | 0.995 |
| GR: Greece | 31 | 657 | 2.25 | 1.55 | 3.26 | 0.0000198 |
| HK: Hong Kong | 4 | 36 | 2.68 | 0.89 | 8.09 | 0.0795 |
| HN: Honduras | 0 | 6 | 0.00000261 | 0 | Infinity | 0.994 |
| HR: Croatia | 20 | 157 | 4.27 | 2.6 | 7 | 8.84E-09 |
| HT: Haiti | 0 | 1 | 0.00000345 | 0 | Infinity | 0.997 |
| HU: Hungary | 46 | 192 | 2.76 | 1.75 | 4.37 | 0.0000139 |
| ID: Indonesia | 0 | 11 | 0.000000389 | 0 | Infinity | 0.99 |
| IE: Ireland | 13 | 673 | 0.496 | 0.283 | 0.87 | 0.0145 |
| IL: Israel | 11 | 328 | 0.954 | 0.516 | 1.76 | 0.88 |
| IN: India | 9 | 692 | 0.301 | 0.155 | 0.585 | 0.000405 |
| IR: Iran | 11 | 130 | 2.34 | 1.24 | 4.41 | 0.00894 |
| IS: Iceland | 0 | 19 | 0.00000122 | 0 | Infinity | 0.987 |
| IT: Italy | 276 | 7389 | 1.28 | 1.11 | 1.46 | 0.000412 |
| JM: Jamaica | 0 | 3 | 0.000000869 | 0 | Infinity | 0.995 |
| JO: Jordan | 0 | 13 | 0.000000379 | 0 | Infinity | 0.989 |
| JP: Japan | 225 | 3584 | 2.3 | 1.97 | 2.67 | 2.77E-27 |
| KE: Kenya | 0 | 2 | 0.000000688 | 0 | Infinity | 0.996 |
| KR: South Korea | 64 | 627 | 2.78 | 2.12 | 3.66 | 2.74E-13 |
| KW: Kuwait | 0 | 81 | 0.00000019 | 0 | Infinity | 0.971 |
| KZ: Kazakhstan | 0 | 2 | 0.000000216 | 0 | Infinity | 0.996 |
| LB: Lebanon | 0 | 47 | 0.00000188 | 0 | Infinity | 0.981 |
| LK: Sri Lanka | 0 | 9 | 0.000000232 | 0 | Infinity | 0.991 |
| LS: Lesotho | 0 | 1 | 0.00000219 | 0 | Infinity | 0.997 |
| LT: Lithuania | 18 | 237 | 1.97 | 1.19 | 3.24 | 0.00788 |
| LU: Luxembourg | 5 | 81 | 0.859 | 0.339 | 2.18 | 0.75 |
| LV: Libya | 0 | 1 | 0.000000384 | 0 | Infinity | 0.997 |
| MA: Morocco | 0 | 8 | 0.000000867 | 0 | Infinity | 0.992 |
| MC: Monaco | 0 | 25 | 0.000000383 | 0 | Infinity | 0.984 |
| MD: Moldova | 0 | 1 | 0.000000382 | 0 | Infinity | 0.997 |
| MK: Macedonia | 0 | 1 | 0.000000916 | 0 | Infinity | 0.997 |
| MM: Myanmar | 0 | 2 | 0.00000179 | 0 | Infinity | 0.996 |
| MT: Malta | 0 | 7 | 0.00000143 | 0 | Infinity | 0.993 |
| MX: Mexico | 8 | 255 | 1.05 | 0.503 | 2.17 | 0.904 |
| MY: Malaysia | 6 | 107 | 1.16 | 0.502 | 2.67 | 0.73 |
| NG: Nigeria | 0 | 2 | 0.00000145 | 0 | Infinity | 0.996 |
| NL: Netherlands | 143 | 3437 | 1.27 | 1.06 | 1.52 | 0.00854 |
| NO: Norway | 90 | 447 | 4.55 | 3.55 | 5.83 | 7.79E-33 |
| NP: Nepal | 0 | 13 | 0.00000107 | 0 | Infinity | 0.99 |
| NZ: New Zealand | 0 | 121 | 0.00000107 | 6.67E-299 | 1.71E+286 | 0.968 |
| PA: Panama | 8 | 28 | 3.29 | 1.45 | 7.47 | 0.00447 |
| PE: Peru | 0 | 49 | 0.000000647 | 0 | Infinity | 0.979 |
| PH: Philippines | 0 | 34 | 0.000000434 | 0 | Infinity | 0.982 |
| PK: Pakistan | 0 | 19 | 0.000000555 | 0 | Infinity | 0.987 |
| PL: Poland | 40 | 3443 | 0.472 | 0.343 | 0.65 | 0.00000407 |
| PR: Puerto Rico | 0 | 347 | 0.00000161 | 2.19E-180 | 1.19E+168 | 0.948 |
| PT: Portugal | 29 | 2121 | 0.509 | 0.35 | 0.74 | 0.000406 |
| QA: Qatar | 2 | 10 | 9.46 | 2.02 | 44.3 | 0.00432 |
| RO: Romania | 13 | 325 | 0.982 | 0.557 | 1.73 | 0.951 |
| RS: Serbia | 0 | 56 | 0.000000773 | 0 | Infinity | 0.978 |
| RU: Russia | 8 | 101 | 1.2 | 0.541 | 2.66 | 0.654 |
| SA: Saudi Arabia | 0 | 37 | 0.000000601 | 0 | Infinity | 0.982 |
| SE: Sweden | 61 | 1993 | 0.715 | 0.549 | 0.929 | 0.0122 |
| SG: Singapore | 0 | 34 | 0.0000018 | 0 | Infinity | 0.983 |
| SI: Slovenia | 13 | 276 | 1.29 | 0.725 | 2.3 | 0.384 |
| SK: Slovak Republic | 1 | 704 | 0.0376 | 0.00527 | 0.268 | 0.00106 |
| SS: South Sudan | 0 | 1 | 0.000000536 | 0 | Infinity | 0.997 |
| SV: El Salvador | 8 | 2 | 39.9 | 6.67 | 239 | 0.0000541 |
| SZ: Eswatini | 0 | 2 | 0.000000451 | 0 | Infinity | 0.996 |
| TH: Thailand | 4 | 72 | 1.16 | 0.414 | 3.27 | 0.774 |
| TJ: Tajikistan | 0 | 1 | 0.00000023 | 0 | Infinity | 0.997 |
| TN: Tunisia | 0 | 28 | 0.000000642 | 0 | Infinity | 0.984 |
| TR: Türkiye | 27 | 1182 | 0.42 | 0.284 | 0.621 | 0.0000138 |
| TW: Taiwan | 11 | 394 | 0.868 | 0.471 | 1.6 | 0.649 |
| TZ: Tanzania | 0 | 1 | 0.00000242 | 0 | Infinity | 0.997 |
| UA: Ukraine | 1 | 25 | 1.48 | 0.197 | 11.1 | 0.702 |
| UG: Uganda | 0 | 3 | 0.00000127 | 0 | Infinity | 0.995 |
| UM: US Minor Outlying Islands | 0 | 2 | 0.000000194 | 0 | Infinity | 0.996 |
| UY: Uruguay | 0 | 2 | 0.0000125 | 0 | Infinity | 0.997 |
| VE: Venezuela | 0 | 4 | 0.00000178 | 0 | Infinity | 0.995 |
| VN: Viet Nam | 0 | 1 | 0.00000863 | 0 | Infinity | 0.998 |
| ZA: Zambia | 12 | 518 | 0.821 | 0.458 | 1.47 | 0.507 |
| ZW: Zimbabwe | 0 | 2 | 0.00000104 | 0 | Infinity | 0.996 |
